# Signal Status Recognition Based on 1DCNN and Its Feature Extraction Mechanism Analysis

**DOI:** 10.3390/s19092018

**Published:** 2019-04-29

**Authors:** Shuzhan Huang, Jian Tang, Juying Dai, Yangyang Wang

**Affiliations:** 1 Graduate School, Army Engineering University of PLA, Nanjing 210000, China; hsz_221618@163.com (S.H.); wyyoung2013@126.com (Y.W.); 2 School of Field Engineering, Army Engineering University of PLA, Nanjing 210000, China; dinajy2001@126.com

**Keywords:** convolutional neural network, intelligent fault diagnosis, convolution kernel, feature extraction mechanism

## Abstract

In this paper, we construct a one-dimensional convolutional neural network (1DCNN), which directly takes as the input the vibration signal in the mechanical operation process. It can realize intelligent mechanical fault diagnosis and ensure the authenticity of signal samples. Moreover, due to the excellent interpretability of the 1DCNN, we can explain the feature extraction mechanism of convolution and the synergistic work ability of the convolution kernel by analyzing convolution kernels and their output results in the time-domain, frequency-domain. What’s more, we propose a novel network parameter-optimization method by matching the features of the convolution kernel with those of the original signal. A large number of experiments proved that, this optimization method improve the diagnostic accuracy and the operational efficiency greatly.

## 1. Introduction

With the increase of system complexity and the increasing variety of data dimensionality, diagnosis systems need better learning and generalization ability, and diagnosis systems based on knowledge and experience alone may find it difficult to meet the needs of modern intelligent fault diagnosis. Moreover, with the deepening of the research, the forms of input signal data become more and more complex and varied, its objectivity and accuracy may be affected if it is still based on previous experience in the feature extraction stage [1,2,3]. Therefore, intelligent fault diagnosis [4,5] is of great significance. 

**Figure 1:**
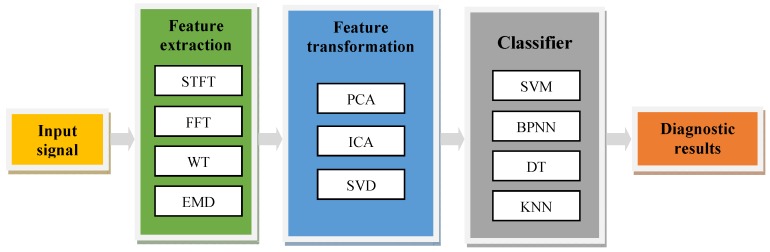
The existing process of intelligent fault diagnosis.

The existing process of intelligent fault diagnosis is shown in Figure 1, which is mainly composed of several independent models, such as feature extraction, feature transformation and classifier. In the feature extraction process, there are many signal processing methods such as: Fourier Transform (FT), Short-time Fourier Transform (STFT), Wavelet Transform (WT), Empirical Mode Decomposition (EMD), etc. By making some transformation of the time-series signal, the above methods can extract the features which are more conducive to classification and diagnosis [6,7]. Such a diagnostic method is effective for the typical characteristics of simple machinery, because its state signals often have obvious frequency characteristics. However, for complex machinery, it is difficult to judge the fault mode based on frequency characteristics or time-frequency characteristics alone depending on human experience. At the same time, some methods still have many shortcomings. For example, FT has spectrum leakage and is not suitable for non-stationary signals because the transformed frequency features do not have any time domain information. STFT can realize local time-frequency analysis, but once the window function is selected, the size and shape of the window is fixed. It can’t adjust adaptively according to the time-frequency characteristics of the signal. WT [8] is a well-known “mathematical microscope”, which can realize the local analysis time-frequency and is more adaptive to the analysis of time-varying signals. However, the selection of the wavelet basis function, the determination of the layers of decomposition are still difficult during the application of wavelet. EMD is an adaptive time-frequency processing method proposed by Huang et al., which is especially suitable for non-linear and non-stationary signals, but it is essentially an empirical signal decomposition without precise formula definition, so it is difficult to analyze theoretically [9,10].

The features obtained by the above signal processing methods are usually of high dimensionality or large size, which is not conducive to the subsequent pattern recognition, so many researchers have tried to improve the identifiability of features by using feature transformation methods such as sparse process, data dimension reduction. The most studied methods include Independent Component Correlation Algorithm (ICA) [11,12], and Singular Value Decomposition (SVD) [13].

The classifier is used to classify the results of feature extraction and transformation, Support Vector Machine (SVM) and Back Propagation Neural Network (BPNN) are commonly used. For example, Cheng et al. [14,15] extracted the feature of roller bearing vibration signals by an improved Local Mean Decomposition (LMD) method, and used SVM as classifier to diagnose bearing faults. Yuan et al. [16] extracted the eigenvalue of hydraulic oil pressure as the input of a BP network to obtain diagnosis results.

For simple faults of simple machines, this kind of step-by-step method is not bad. However, as the systems become more and more complex and the dimensionality of the data becomes higher and higher, there will be some new faults. A highly intelligent diagnostic model should have good generalization ability to deal with and identify new faults. Obviously the above methods shown in Figure 1 do not have this kind of ability. More importantly, the traditional feature extraction process is not directly oriented to the fault mode classification. There is no joint optimization between them, that is, the feature extractors and the classifiers are independent of each other. This kind of model will lead to redundancy or errors in the feature extraction. It is not conducive to classification.

Therefore, it is very important to obtain an intelligent diagnosis model, which can not only realize the joint optimization of feature extraction and pattern recognition, but also has a good generalization ability. The newly proposed Deep Learning (DL) model provides the possibility to realize such an idea.

CNN is such a deep learning structure [17,18,19]. It is composed of several convolutional layers and pooling layers to carry out feature extraction and data reduction. Then, the fully connected layer and output layer classify and output the results. Compared with the methods mentioned above, CNN integrates several steps (feature extraction, feature transformation, information fusion, pattern recognition) into one deep structure to realize their joint optimization and complete the intelligent diagnosis, so CNN’s adaptive ability and generalization ability are more excellent [20,21]. Because of these advantages, it has been widely used in many fields [22,23,24], and has achieved many good achievements. For example, in 2012, Krizhevsky et al. [25] used CNN in the ImageNet Large Scale Visual Recognition Challenge, and achieved the best classification. In the field of fault diagnosis, CNN has attracted the attention of many scholars and been applied to intelligent fault diagnosis. There are three kind of studies:

(1) The first kind is to transform the signals into the frequency domain. To complete the classification, the frequency features are extracted by CNN. For example Janssen et al. [26] from Ghent University in Belgium used CNN to diagnose bearing and gear faults in the gearbox. They applied a Discrete Fourier Transform (DFT) to transform vibration signals into the frequency domain, and trained the CNN with labeled samples to complete the diagnosis. The accuracy of fault diagnosis was increased by 6%. Obviously, in this kind of researches, CNN just worked as the classifier, its excellent multi-layer feature extraction and abstraction ability were underutilized.
(2) The second kind is to use the images of time-series signals as the input of 2DCNN. Wen et al. [27] used images of bearing vibration signals as the input of CNN, and the diagnosis accuracy was above 95%. Hoang et al. [28] adopted a deep CNN structure in the fault diagnosis of rolling bearings, which had higher accuracy and robustness, even in noisy environments. Actually, this kind of method treats the fault diagnosis problem as an image recognition problem. However, complex machines often work in large noisy environments. Their state signals often contain various frequency components. If the fault diagnosis is only based on the shape of the time-series signal, instead of digging out the deeper features, whether it can maintain a good identification ability when faced with the complex signal of complex machine is questionable.
(3) The third kind is to convert time-series signals into two-dimensional time-frequency diagrams, and use them as the input of 2DCNN for diagnosis. For example, Guo et al. [29] used the transform result after continuous WT as the input matrix of CNN to diagnose the fault of rotating machinery, and achieved a good diagnosis result. Wang et al. [30] preprocessed the original signal with STFT to obtain the time-frequency diagram, and then used CNN to adaptively extract the time-frequency features to complete diagnosis. This kind of methods adds a time-frequency step before CNN, but as mentioned earlier, the current commonly used time-frequency transformation methods may cause some degree of distortion of the original signal, such as the loss of useful features, the appearance of false features, signal distortion and so on.

The above three methods are all based on 2DCNN, so many researchers prefer to 2DCNN because of its successful application in the field of image recognition. For the computer, an image is a two-dimensional matrix, while the real-time state signal (vibration and pressure signal, etc.) during the operation of machine is usually a one-dimensional vector. Therefore, some scholars have tried to construct 1DCNN to realize fault diagnosis. For example, Turker et al. [31] tested the current of a motor, and used 1DCNN to realize real-time state monitoring and fault diagnosis. Peng et al. [32] used 1DCNN to diagnose the faults of HST wheelset bearings with vibration signals, and achieved good results. For one dimensional time-series signal, compared to 2DCNN, 1DCNN has a better ability of feature extraction because of two advantages [33]: firstly, there are no signal processing methods before CNN, such as FT, WT. The original time-series signal is input directly, which can guarantee the authenticity of the input. Secondly, 1DCNN only performs one-dimensional convolution, which makes the structure simpler and the parameters fewer. Therefore, it can save computing resources and time, which makes it more conducive to real-time monitoring of machines.

Like 2DCNN, 1DCNN will also face with the problem of how to determine the network structure and set the parameters. The number of layers, as well as the size and number of convolution kernels will greatly affect the feature extraction results. Focusing on how to optimize the network performance, some scholars have carried out some relevant studies on 2DCNN. For example, Li et al. [34] proposed a complex convolution kernel under the two-dimensional structure, and gave the suggestions on how to set the convolution kernels through comparative experiments. He et al. [35] explored the relationship between network depth, width and size of convolution kernel through a large number of experiments to optimize the network structure. However, these studies are insufficient. Firstly, the summary of optimization experience based on a large number of experiments, and the repeatability is poor. Secondly, when faced with different diagnostic problems, because there is almost no in-depth analysis on the training results and the principle of feature extraction, their conclusions are difficult to reference.

In CNN, the most crucial structure is the convolution layer compose of several convolution kernels. It is worth noting that the frequently used signal processing methods, such as FT and WT, are essentially kernel operations based on the kernel. These methods select the kernels manually, while the kernels of CNN are acquired by adaptive learning. Obviously, for one-dimensional time-series signals, the convolution results of 1DCNN are more interpretable than those of 2DCNN. Therefore, it is necessary to analyze the training results and layer-by-layer transformation results of 1DCNN deeply, which is not only conducive to a better explanation of its diagnostic mechanism, but also helpful to guiding the optimization of network structure and parameters.

This paper attempts to construct one-dimensional CNN oriented to fault diagnosis, and uses one-dimensional time-series signals as its input. Through in-depth analysis the convolution (feature extraction) mechanism, it attempt to provide a useful suggestion for how to select the number of convolution kernels, and thus to optimize the network performance. The main contents and research ideas of this paper are as follows: Section 2 introduces the network structure and advantages of 1DCNN. Section 3 creates the simulation signals to analyze the feature extraction mechanism. Section 4 analyzes the convolution kernel and convolution result of the bearing state signal, proposes a parameter optimization method and verifies it by experiments. Section 5 presents the conclusions.

## 2. The One Dimensional Convolutional Neural Network Model

### 2.1. The Structure of 1DCNN

**Figure 2:**
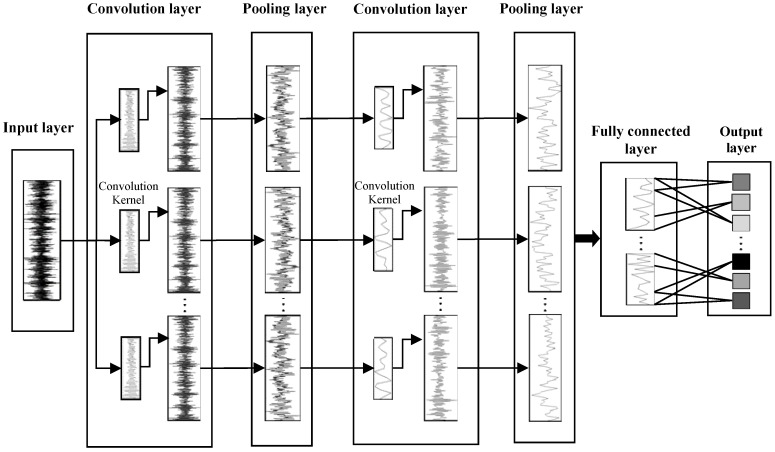
The structure of 1DCNN.

The 1DCNN structure is shown in Figure 2. The time-series signals are used as the input of the input layer. Then the convolutional layers and pooling layers are used for feature extraction and sparse processing layer by layer, where each convolution layer is composed of several convolution kernels, and the convolution kernels in the same layer have the same size. The pooling layers adopt the method of average pooling, after that, the fully connected layer classifies the results. 

#### 2.1.1. Convolution Layer

In the convolution layer, the 1DCNN carries out the convolution operation on the local area of the input signals to generate the corresponding one-dimensional feature maps, and different convolution kernels extract different features from the input signal, respectively. Each convolution kernel detects specific features at all locations on the input feature map, to fulfill the weight-sharing on the same input feature map. This characteristic of local connectivity and weight-sharing effectively reduces the complexity of the network and the number of training parameters. If the L layer is a convolution layer, the formula for the one-dimensional convolution layer is:

(1) $$x_{j}^{l}=f\left(\sum_{i=1}^{M}x_{i}^{l-1}*k_{ij}^{l}+b_{j}^{l}\right)$$

where *k* represents the convolution kernels, *j* denotes the number of kernels, *M* represents the channel number of input $x^{l-1}$. *b* is the bias corresponding to the kernel, *f* () is the activation function and ∗ is the convolution operator.

#### 2.1.2. Pooling Layer

In the down sampling phase, after the convolution layer, the number of feature maps increases, resulting in the expansion of the data dimensionality, which is not conducive to calculation. Therefore, the average pooling or max pooling method is used to process each feature map at this stage. Average pooling calculates according to the size of the predetermined pooling window, and the maximum pool method selects the maximum parameter within the range of the predetermined window as the output value.

#### 2.1.3. Fully Connected Layer

The fully connected layer of the neuron nodes are connected to all the neuron nodes in the feature map output from the previous layer, and the activation function is the Softmax function. If the final pooling layer is *l* + 1 and its output is given to the fully connected layer, the output of the fully connected layer is:

(2) $$h\left(x\right)=f\left(w^{l+1}\cdot x^{l+1}+b^{l+1}\right)$$

where *w* denotes the weight and *b* denotes the bias.

### 2.2. Network Training

**Figure 3:**
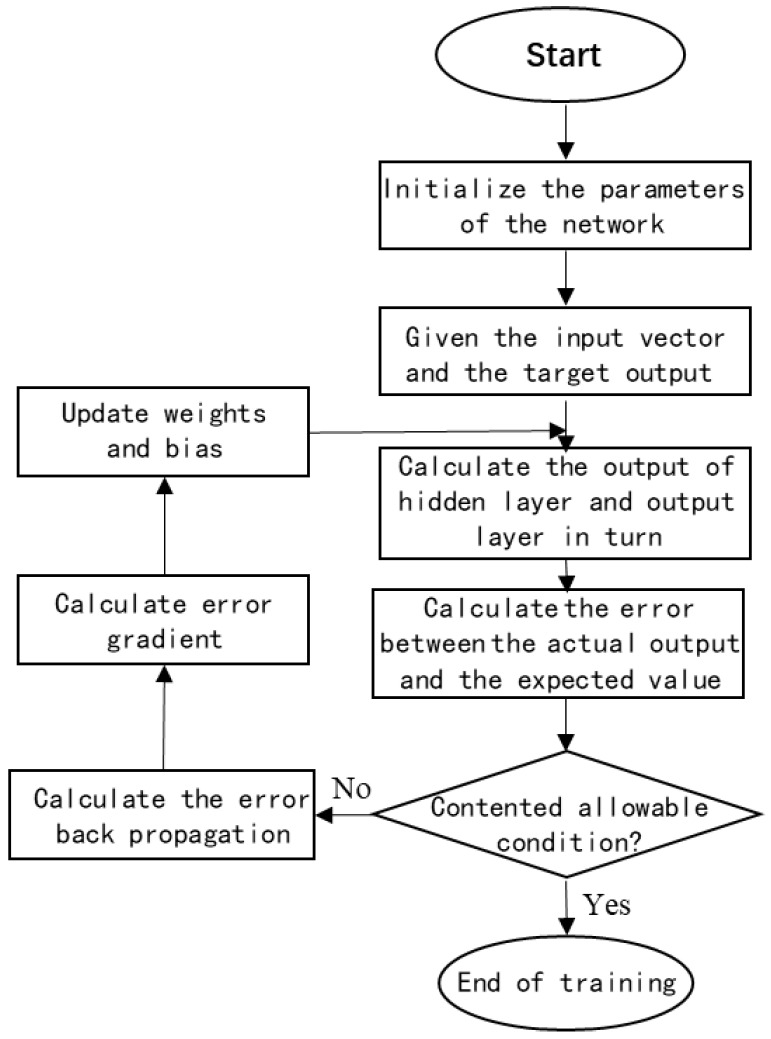
The process of network training.

In the process of network training [36], the parameters such as weight and bias are initialized, The input is propagated forward through the convolution layer, pooling layer and fully connected layer to obtain the output value. Then the error between the output value and the expected value is calculated, and the errors of the fully connected layer, the pooling layer and the convolution layer are obtained successively, and after that, the error gradient is calculated. The training is completed when the weights and bias are updated until the admissible conditions for errors are met. The process is as shown in Figure 3.

## 3. Feature Extraction Mechanism Analysis

### 3.1. The Pattern Recognition of Simulation Signal

#### 3.1.1. Creation of Experimental Samples

The following is a simple time-series signal classification problem to verify the feasibility of the study, four types of signals are generated under the Matlab environment. The signal can be expressed as:

(3) $$Y_{i}=e^{-0.05}\left[A_{i}\sin\left(\omega_{i1}t+\varphi_{i1}\right)+B_{i}\sin\left(\omega_{i2}t+\varphi_{i2}\right)+C_{i}\sin\left(\omega_{i3}t+\varphi_{i3}\right)\right]+u(t)$$

where *u*(*t*) is the white Gaussian noise series with 10 dB; and $e^{-0.05}$ represents the damping characteristics of system. The parameter-setting standards for each type of signal parameter are shown in Table 1.

**Figure 4:**
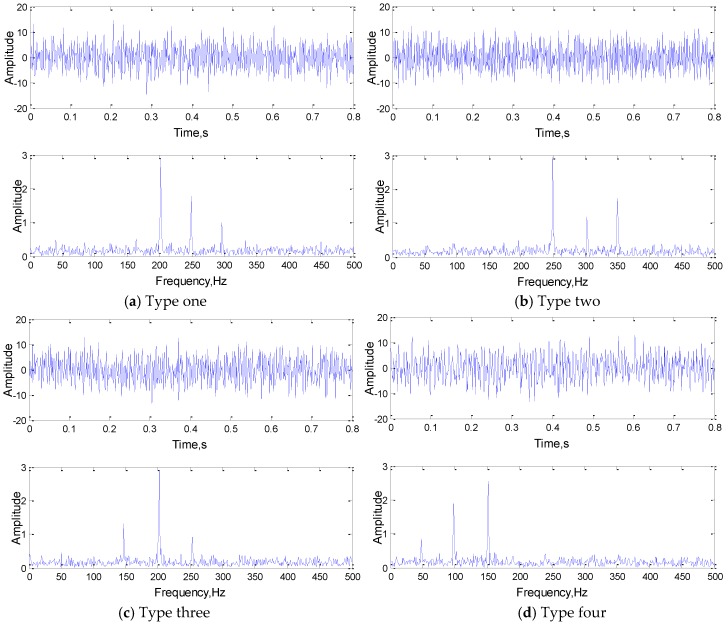
The time and frequency domain diagrams of the simulation signals.

In order to ensure the diversity and the otherness between different categories of samples, each type of signal consists of different frequency components. Meanwhile, the frequency components in each sample are subject to uniform distribution within the certain range, while the phase angle is generated randomly between 1 and 100, which can ensure the correctness and diversity of the same type of signals. To increase the complexity and reduce the overfitting phenomenon of the network, the attenuation coefficient and Gaussian white noise are added to the simulation signal. Their time domain and frequency domain diagrams are shown in Figure 4.

#### 3.1.2. Parameter Setting of the 1DCNN

The network structure consists of an input layer, two convolution layers, two pooling layers, a fully connected layer and an output layer, and each layer is provided with several convolution kernels. In order to simplify the expression, *N_i_*(*M_i_*)–*N_i_*_+1_(*M_i_*_+2_) is used to describe the network structure and related parameters of the convolution kernel, where *N* represents the number of convolution kernels, *M* represents the size of convolution kernels, and *i* represents the convolution layer. The learning rate is 1, and the system updates the weights every 10 samples, and the maximum number of iterations is 1000.

#### 3.1.3. Training Results and Preliminary Conclusions

In the experiment, we used those four types of signals, 125 samples for each type of signal (100 training samples, 25 test samples). The feature extraction and classification ability of 1DCNN was tested based on the simulation signals, and the results are shown in Table 2. 

We can conclude that the 1DCNN has good classification ability to classify those four kinds of signals. The following two preliminary conclusions can obtain by analysis of the training results: the selection of the number and size of convolution kernels in each layer will greatly affect the operation efficiency and training results of the network. As can be seen from Table 2, the number of convolution kernels has a greater impact on the accuracy and efficiency of training. More convolution kernels are not better, when the training accuracy rate can meet the requirements of classification. With the increase of the number of convolution kernels, the training times will be longer and the computational resources consumption will increased, and excessive convolution kernels will reduce the network training accuracy. However, too few convolution kernels will cause the training and the correct classification to fail. It is very necessary to configure the number and size of convolution kernels reasonably.

**Figure 5:**
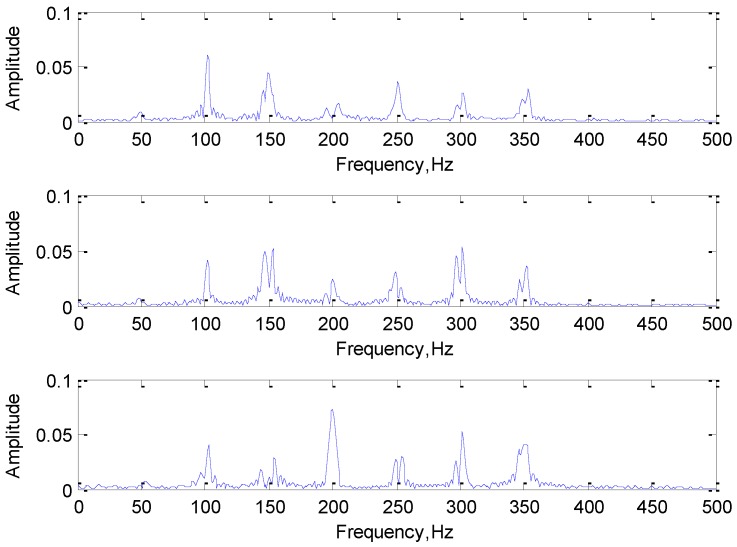
The frequency domain analysis of the three convolution kernels.

Figure 5 shows the FFT transformation results of the three convolution kernels of the first convolution layer in a randomly selected 1DCNN network (3(301)-3(301)), and their classification effect must be good. According to the Figure 5, the convolution kernel is analogous to a digital filter in the 1DCNN. The convolution kernels in the network contain typical frequency components of the original input signal, which indicates that the training process causes the parameters of the convolution kernel to gradually approximate a digital filter. After convolution with the kernels, their typical corresponding frequency components will retain as the input of the next layers.

### 3.2. The Role of Convolutional Kernel

#### 3.2.1. The Evolution of the Convolution Kernel

**Figure 6:**
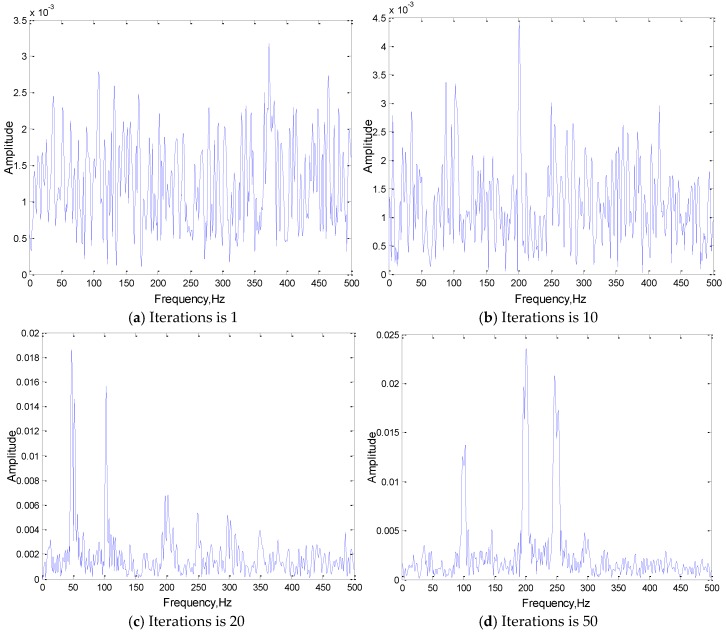
The frequency domain analysis of convolution kernel in different iteration stages.

The network with the best training effect, whose structure is 3(301)-3(301), is used to extract the convolution kernel of the first convolution layer in different training iteration stages. The signal characteristics of the extracted convolution kernel and its own changing trend are analyzed in this section. Figure 6 is the frequency domain analysis of the convolution kernel in different iteration stages.

**Figure 7:**
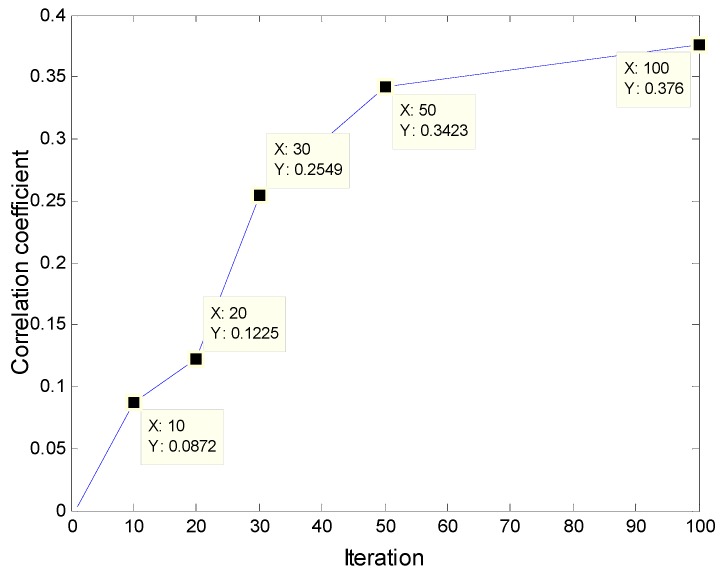
The correlation coefficient between the convolution kernel and the original signal in different iteration stages.

Figure 7 is the changing process of the correlation coefficient between the convolution kernel and the original signal with the number of training iterations:

The following conclusions can be drawn, with adaptive learning training, the frequency components of the convolution kernel that are the same as the corresponding original signal will be obtained, and it can help to highlight the main features (frequency components) of the input original signal. As the number of iterations increases, the correlation coefficient between the convolution kernels and the input signals is increasing. After the convergence of iterative training, the convolution kernel shows a good correlation with the original signal, which indicates that in the adaptive learning process of the convolution kernel, the feature frequency of the original signal gradually appears in the convolution kernels, and its correlation with the original signal is constantly increasing. In the convolution stage, the more similar the form of the kernel function is to that of the original signal, the better the effect of convolution feature extraction will be.

#### 3.2.2. Convolution Results Analysis 

**Figure 8:**
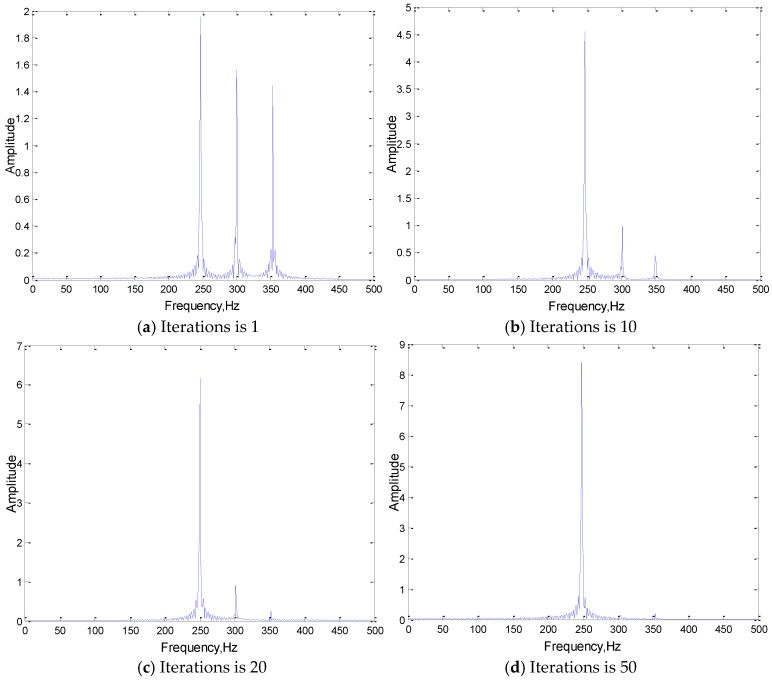
The frequency domain analysis of convolution results.

In order to explore the role of convolution kernels further, the output results after the feature extraction (convolution) of the first layer convolution kernel of the original signal are extracted, and the convolution results are analyzed in frequency domain. The analysis results are shown in Figure 8.

As can be seen from Figure 8, convolution kernels do play the role as a frequency domain filter in the feature extraction process of 1DCNN. As the training iteration increases, the main frequency components are extracted gradually as the “feature vectors” for feature extraction and classification, and the rest of the atypical frequency components in the input are filtered out.

### 3.3. Collaborative Optimization for Classification Problems

#### 3.3.1. The Qualitative Analysis

**Figure 9:**
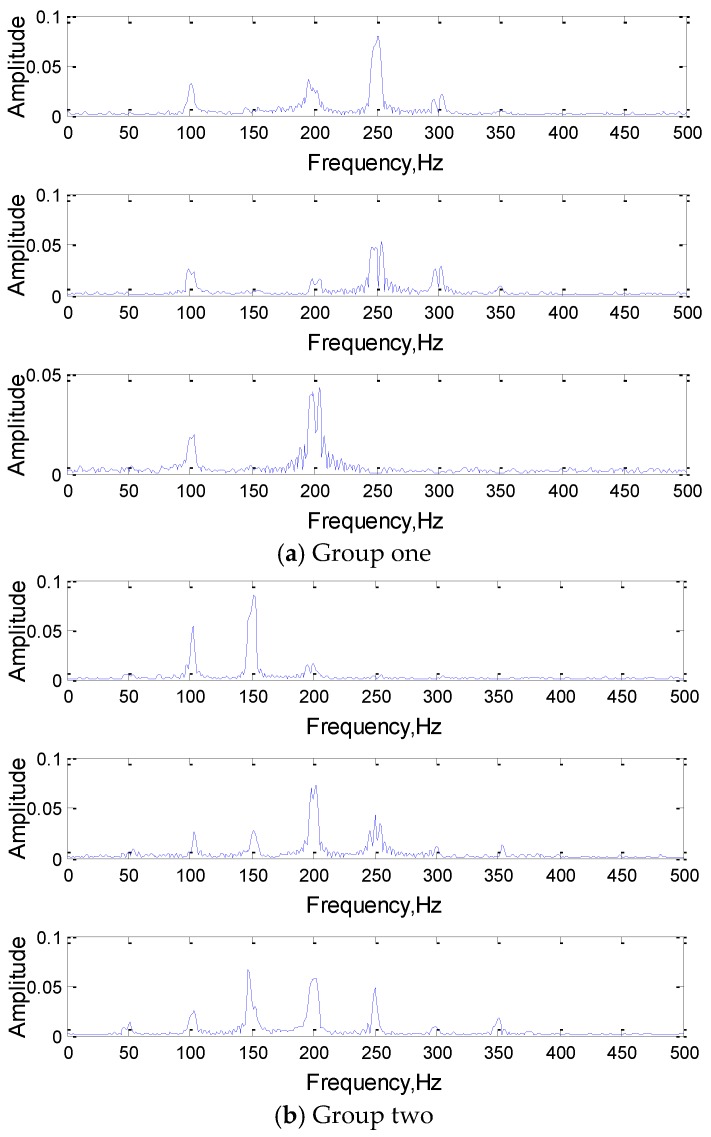

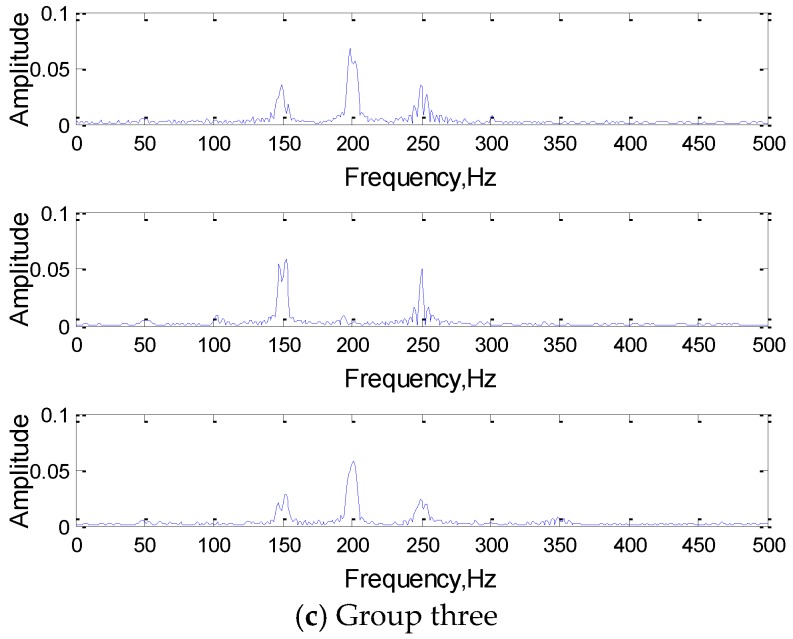
The frequency domain analysis of three convolution kernels in a group.

To comprehend the working mechanism of 1DCNN deeply, a set of convolution kernels in the same layer are analyzed in this section. Since the number of convolution kernels set by the network is 3, that is, there are three convolution kernels convolving the same input simultaneously, and the extracted features of the three convolution kernels will jointly act on the classification and recognition of the signal. Frequency domain analysis of three convolution kernels in the same group as shown in Figure 9.

As we can see from the Figure 9, the features of the input signal were extracted by a set of three convolution kernels (filter), the characteristic frequency components of the input signal were obtained in the convolution kernels. The frequency components of the same set were also different, which met from different perspectives to express the input signal features, and realize the classification of the signal together. 

It is worth noting that since only some key feature components need to be extracted to realize the expression and classification, the single convolution kernel will not extract all the frequency components of the input signal as the feature vectors. This intelligent feature extraction mode of the network greatly reduces the redundancy of feature extraction and improves the efficiency of feature extraction and classification.

#### 3.3.2. The Quantitative Analysis

Meanwhile, in order to analyze the relationship between kernels quantitatively, the correlation between the three groups of convolution kernels in Figure 9 is analyzed, and the correlation coefficient matrix is shown in Table 3, Table 4 and Table 5, Table 6 shows the average of the correlation coefficient between the convolution kernels after a total of 36 training instances.

As can be seen from these tables, at the end of the training, the correlation between the same set of convolution kernels is very small, that is, each convolution kernel extracts part of the input signal features, which are independent of each other, and multiple features jointly complete the feature extraction of the input signal. The same set of convolution kernels cooperates on the recognition and classification of signals when facing the classification problem, realizing the feature sparsity and reducing the redundancy between features.

Above all, in the 1DCNN, since the convolution kernel is equivalent to the frequency domain filter, the same set of convolution kernels will represent the original signal from different perspectives, they can extract the features of the original signal, and fuse these features to realize the abstract expression of the original signal. Then, when we need to set the convolution kernel parameter, the number of the original signal frequency component and the number of convolution kernel could be corresponding, so that each convolution kernel (filter) can correspond to express some typical frequency component. In this way, each feature component of the original signal can express fully, the insufficient or redundant feature expression in the feature extraction process will be reduced, and the classification recognition rate and network computing speed will be improved.

## 4. Fault Diagnosis of Bearing Vibration Signals

### 4.1. Data Preparation

#### 4.1.1. Data Description

In the actual diagnosis process, because the mechanical operation will be affected by many factors, its vibration signal is often non-stationary, and contains a large amount of noise. In order to verify the relationship between 1DCNN performance and signal frequency components and the number of network convolution kernels presented in this paper, validation experiments were carried out on the bearing state signal of the public data set from Case Western Reserve University (CWRU). The object of this test is the drive-end bearing, which is a SKF6205 deep groove ball bearing. The single-point damage of the bearing was caused by EDM, and the sampling frequency is 12 kHz. There are four states of bearings: normal, rolling element damage, inner ring damage, outer ring damage, with a damage diameter of 0.007 inches. 

#### 4.1.2. Data Augmentation

**Figure 10:**
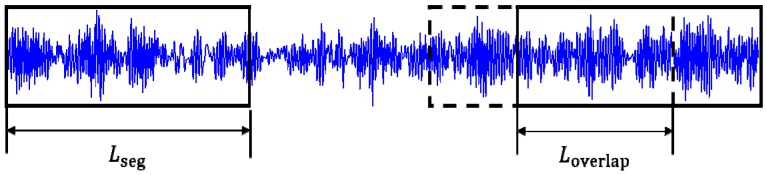
Data augmentation.

Since the bearing vibration signal is one-dimensional, considering that the main information of the signal contains the impact generated in different states, we introduce a sliding segmentation method as shown in Figure 10 to get more training samples. It can also maintain the continuity of signals, which is conducive to real-time fault diagnosis, so that the model can be more robustness as far as possible, and effectively reduce the overfitting phenomenon. The segmentation calculation formula is as follows:

(4) $$n=\left(N-1\right)\times \left(L_{seg}-L_{overlap}\right)+L_{seg}$$

where *n* is the number of sampling points of vibration signals, $L_{overlap}$ is the length of overlapping samples, $L_{seg}$ is the length of each segment, and *N* is the number of segments.

There are about 100,000 sampling points for each type of the bearings state signals. The first 80,000 points are used as the training samples. The sample length is 1024 and the overlapping length is 226. Therefore, the number of training samples is 400. The remaining points used as the test samples. In order to test the ability of robust and generalization, the overlapping sampling method was not used on 100 test samples.

#### 4.1.3. Data Analysis

**Figure 11:**
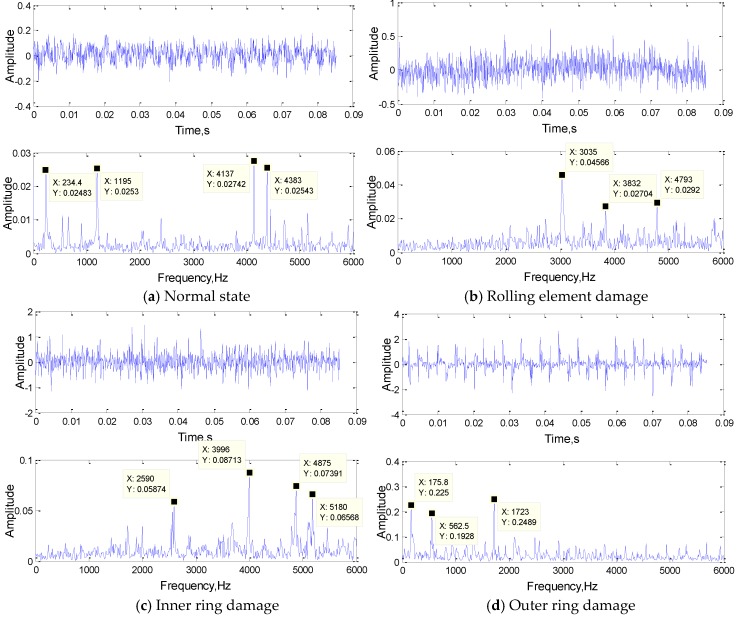
The time-domain signals of four kinds of state signals and their FFT results.

Before training, the original signal was analyzed preliminarily, the time-domain signals of four kinds of state signals and their FFT results are shown in Figure 11.

### 4.2. Feature Extraction Mechanism Analysis

#### 4.2.1. Convolution Result Analysis

**Figure 12:**
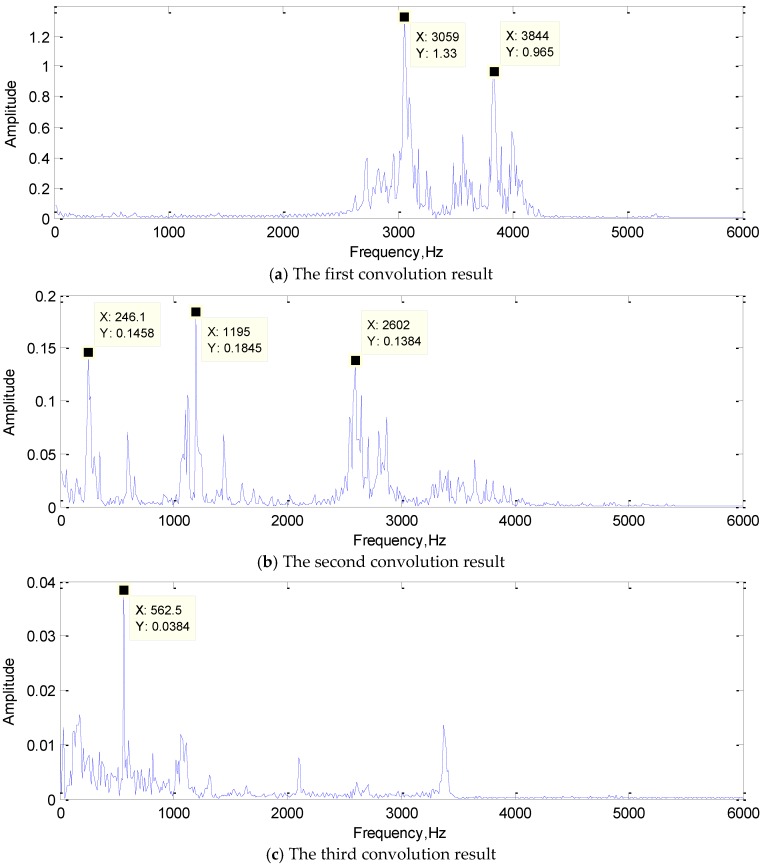
The FFT of the convolution result.

The convolution result is the feature extraction result. It is not only the output of the convolution layer, but also the input of the subsequent layer. It represents the features extracted by the convolution kernel adaptively learned from training samples. By visualizing the convolution results and analyzing the frequency components, the function of each convolution kernel in real-time fault diagnosis could be revealed. In addition, it is also clear that which features in the signal are selected by the convolution kernel as the classification “standard” to represent the original signal. This may also provide new ideas for some artificial feature extraction methods. The FFT of the convolution results are shown in Figure 12.

In Figure 12a, the frequencies of 3035 Hz and 3832 Hz were extracted as the features; they are both the typical frequency components of the rolling element damage signal. In Figure 12b, 246.1 Hz and 1195 Hz were the typical frequency components of the normal state signal, and 2602 Hz was also approximately the same frequency as the inner ring damage signal. The frequency of 562.5 Hz is the typical frequency components of the inner ring damage signal as shown in the Figure 12c. Obviously, the convolution kernel can filter out the frequency components of the original signal and retains several typical frequency components as the result of feature extraction. The first convolutional layer realized the primary feature extraction, and the convolution result would send to the subsequent network as input for further feature extraction.

#### 4.2.2. Convolution Kernel Analysis

**Figure 13:**
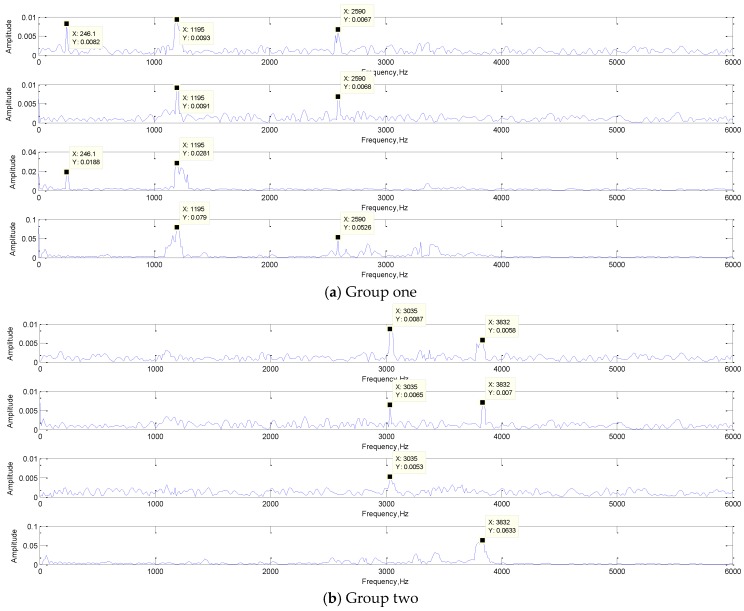
The FFT results of the convolution kernel.

Convolution kernel plays a key role in feature extraction. The number and size of convolution kernel will greatly influence the effect of feature extraction. Therefore, in this section, the convolution kernel of the first layer of the network was analyzed. The cooperative classification of multiple convolution of the same input signal was analyzed, and the relationship between the number of convolution kernels and the feature extraction effect was obtained. The FFT results of the convolution kernel are shown in Figure 13.

As shown in the Figure 13, the frequency component of the original signal appeared in the convolution kernel. It acquired some features of the original signal in frequency domain adaptively by training. At the same time, in the process of feature extraction, the convolution kernels have different frequency components, so they played different roles independently, and the feature extraction results of the signal were different. A set of convolution kernels extracted the input signal features from different perspectives, and the extracted results acted on the classification of signals together, to realize the intelligent feature extraction. Because of the joint optimization effect of convolution kernel, CNN has excellent feature extraction ability.

### 4.3. Network Optimization

#### 4.3.1. Optimization Method

Through the above analysis, the correctness and feasibility of the study was proved, and it provided a new idea for one-dimensional network optimization. In order to fully express each feature component of the original signal and avoid the over-fitting phenomenon caused by feature extraction redundancy, when setting the network parameters, the number of convolution kernels must correspond to the number of main frequency components of the input signals. According to the Figure 11, the number of main frequency components of the bearing normal state signals and inner ring fault signals are both 4, and the number of main frequency components of the rolling element fault signals and the outer ring fault signals are both 3. Therefore, according to the optimization method, the number of network training convolution kernels is 4. As the complexity of the signal increases, the experiment adopted the network structure of three convolution layers. The number of convolution kernels in each layer was more than that in the previous layer, and the size of convolution kernels in each layer is decreasing, to realize the abstract expression of signal from low resolution to high resolution. By using the parameter optimization method, we constructed a optimized 1DCNN which structure is 4(301)-8(163)-16(80).

#### 4.3.2. Contrast Experiment

In order to verify the effectiveness of the proposed method, a network of other structures was constructed and compared with the optimized network. The learning rate is 1, and the batch-size is 10, and the maximum iteration is both 2000. The comparison results are shown in Table 7.

As can be seen from the Table 7, compared with the network with more convolution kernels, the optimized network 4(301)-8(163)-16(80) consumed the shortest training time when the accuracy met the diagnostic requirements. However, the accuracy of the network with less convolution kernel will decrease, and the network with less convolution kernels will often fail to converge, leading to training failure. The following conclusions can be drawn: for the bearing signals with complex signal components, the deepening of the network layer can make them have better feature extraction and abstract expression ability, and can improve the accuracy of fault signal recognition. More importantly, if the number of convolution kernels are too small, feature extraction will be insufficient or even unable to extract the main features, leading to the failure of correct classification. With the increase of the number of layers and the number of convolution kernels, the abstract expression ability of the network will be improved, but the consumption of computing resource will be greatly increased and the training time will be prolonged, which is not conducive to real-time fault diagnosis. Therefore, in the realistic situation of fault diagnosis, the number of convolution kernels should set according to the typical frequency domain components of the input signals, so that the network will have a good feature extraction ability, and at the same time, the operation cost will save and the operation efficiency will improve.

## 5. Conclusions

In this paper, a fault diagnosis method based on 1DCNN is proposed. This method does not need to do any artificial signal processing on the input signals, and effectively avoids the loss of useful information. By visualizing the output results of convolution and the convolution kernels, this paper explore the feature extraction mechanism of convolution kernel, and the main conclusions can be summarized as follows:

- In the 1DCNN, the convolution kernel acts as the frequency domain filter. Its signal feature form will be similar to the input signals after convolution with the input signals, and the main frequency components of input signals will be extracted. Moreover, the same set of convolution kernels extract the features of input signals from different perspectives, and the extraction results act on the classification together, and the collaborative optimization of signal classification is achieved. Through the study of its convolutional mechanism, it explains why 1DCNN has such a good feature extraction ability.
- The network parameters (the number of convolution kernels) can be optimized by analyzing the frequency domain characteristics of the input signals. The optimization method consists of two steps: firstly, preliminary analysis the original signal by signal processing methods (FFT, STFT), and counting the number of the typical frequency components. Secondly, to express the characteristics of the input signals and improve the network efficiency greatly, and save computing resources, the number of convolution kernels should correspond to the number of typical frequency components of the input signal as far as possible.

## Figures and Tables

**Table 1:** The signal parameter setting criteria.

Type/*i*	$\mathrm{Amplitude}/A_{i}$ $,\;B_{i}$ $,\;C_{i}$	$\mathrm{Frequency}/\omega_{ij}$	$\mathrm{Phase}/\varphi_{ij}$
1	$A_{1}$ = 6, *B*_1_ = 4, *C*_1_ = 2	$\omega_{11}$~u(197,203) $\omega_{12}$~u(247,253) $\omega_{13}$~u(297,303)	$\varphi_{1j}$ = rand[1,100]
2	$A_{2}$ = 6, *B*_2_ = 2, *C*_2_ = 4	$\omega_{21}$~u(247,253) $\omega_{22}$~u(297,303) $\omega_{23}$~u(347,353)	$\varphi_{2j}$ = rand[1,100]
3	$A_{3}$ = 4, *B*_3_ = 6, *C*_3_ = 2	$\omega_{31}$~u(147,153) $\omega_{32}$~u(197,203) $\omega_{33}$~u(247,253)	$\varphi_{3j}$ = rand[1,100]
4	$A_{4}$ = 2, *B*_4_ = 4, *C*_4_ = 6	$\omega_{41}$~u(47,53) $\omega_{42}$~u(97,103) $\omega_{43}$~u(147,153)	$\varphi_{4j}$ = rand[1,100]

**Table 2:** Training results of the simulation signal.

Convolution Kernel Parameter	Average Accuracy/%	Number of Iterations	Training Time per Iterations/s	Total Time/s
1(301)-1(301)	Failure	Failure	0.92	\
3(301)-3(301)	100	63	3.4	214.2
4(301)-4(301)	100	82	5.1	418.2
8(301)-8(301)	95	92	13.7	1260.4
16(301)-16(301)	85	124	46.7	5790.8

**Table 3:** The correlation coefficient between the three kernels of the group 1.

	$K_{11}$	$K_{12}$	$K_{13}$
$K_{11}$	1	0.0201	0.0948
$K_{12}$	0.0201	1	0.1059
$K_{13}$	0.0948	0.1059	1

**Table 4:** The correlation coefficient between the three kernels of the group 2.

	$K_{21}$	$K_{22}$	$K_{23}$
$K_{21}$	1	0.0548	0.0930
$K_{22}$	0.0548	1	0.0852
$K_{23}$	0.0930	0.0852	1

**Table 5:** The correlation coefficient between the three kernels of the group 3.

	$K_{31}$	$K_{32}$	$K_{33}$
$K_{31}$	1	0.0337	0.0745
$K_{32}$	0.0337	1	0.1570
$K_{33}$	0.0745	0.1570	1

**Table 6:** The average correlation coefficient of the convolution kernels.

	${\bar{K}}_{1}$	${\bar{K}}_{2}$	${\bar{K}}_{3}$
${\bar{K}}_{1}$	1	0.0373	0.0641
${\bar{K}}_{2}$	0.0473	1	0.0138
${\bar{K}}_{3}$	0.0641	0.0138	1

**Table 7:** Training results of the bearing vibration signals.

Convolution Kernel Parameter	The Accuracy of Training Data/%	The Accuracy of Test Data/%	Training Time per Iterations/s	Total Time/s
2(301)-4(301)	Failure	Failure	0.8	\
3(301)-6(301)	75	75	1.7	1854.3
4(301)-8(301)	90	87.5	3.0	1742.6
5(301)-10(301)	77.5	77.5	3.2	2667.0
2(301)-4(163)-8(80)	72.5	72.5	2.0	2137.8
3(301)-6(163)-12(80)	90	87.5	4.2	5548.7
4(301)-8(163)-16(80)	100	100	6.6	5537.4
5(301)-10(163)-20(80)	95	95	9.2	9583.4
6(301)-12(163)-24(80)	100	100	14.2	10,432.7
